# New Metabolic, Digestive, and Oxidative Stress-Related Manifestations Associated with Posttraumatic Stress Disorder

**DOI:** 10.1155/2021/5599265

**Published:** 2021-12-20

**Authors:** Bianca Augusta Oroian, Alin Ciobica, Daniel Timofte, Cristinel Stefanescu, Ionela Lăcrămioara Serban

**Affiliations:** ^1^Socola Hospital Iasi, Bucium 36, Iași, Romania 700282; ^2^Department of Biology, Faculty of Biology, Alexandru Ioan Cuza University, B dul Carol I No. 11 Iasi, Romania; ^3^“Grigore T. Popa” University of Medicine and Pharmacy, 16, Universitatii Street, 700115 Iasi, Romania

## Abstract

Posttraumatic stress disorder (PTSD) represents a pressing and generally invalidating syndrome that is triggered by a terrifying or stressful experience, relying on recurrently reliving the traumatic event feelings associated to it, which is subsequently linked to ongoing activations of stress-related neurobiological pathways and is often associated with neurodegeneration. In this paper, we examine what lies beneath this disorder, reviewing evidence that connects PTSD with a wide array of mechanisms and its intertwined pathways that can lead to the decompensation of different pathologies, such as cardiovascular disease, gastrointestinal ailments, autoimmune disorders, and endocrine diseases. Also, the significance of the oxidative stress in this frame of reference is debated. Thus, knowing and identifying the main features of the distressing experience, the circumstances around it, as well as the neuropsychological and emotional characteristics of people prone to develop PTSD after going through disturbing incidents can offer an opportunity to anticipate the development of potential destructive consequences in several psychological dimensions: cognitive, affective, relational, behavioral, and somatic. We can also observe more closely the intricate connections of the disorder to other pathologies and their underlying mechanisms such as inflammation, oxidative stress, bacterial overgrowth syndrome, irritable bowel syndrome, metabolic disorders, oxytocin, and cortisol in order to understand it better and to optimize the course of treatment and its management. The complex foundation PTSD possesses is supported by the existing clinical, preclinical, and experimental data encompassed in the current review. Different biological systems and processes such as the hypothalamic-pituitary-adrenal axis, sympathetic nervous system, oxidative stress, inflammation, and microbiome suffer modifications and changes when it comes to PTSD; that is why targeted therapies exert tremendous alleviations of symptoms in patients diagnosed with this disorder. Therefore, this implies that PTSD is not restricted to the psychiatric domain and should be viewed as a systemic condition.

## 1. Introduction

In current times, we face a great need to study posttraumatic stress disorder (PTSD) together with targeted intervention strategies—a fact determined by events that transpire all around the world, such as population migration, an increasing number of separated families, and high divorce rates, but also unemployment, a poor socioeconomic status, financial issues, marital problems, abusive relationships, chronic stress, a violent climate in the household, psychological harm, fires, road accidents, and crimes against minors and their families.

Scenarios of extensive stress can inflict harm to both the adult and children, ultimately leading to PTSD, a syndrome commonly linked to different mental afflictions, such as major depressive disorder, anxiety and panic attacks, and several dependencies. Children, in particular, fragile as they are, are more prone to develop posttraumatic stress disorder, being susceptible to a wide range of psychological modifications. Therefore, the severity of the disorder requires a complex approach to the problem [1].

Over time, stress has been perceived from three perspectives: (1) stress acting like a stimulus, or a critical event in life, which triggers the physical and mental reaction to stress, such as anxiety or cardiovascular problems [2]; (2) stress as a psychological and physical reaction to acute or chronic strains [3]; and (3) stress as an ongoing process between the person and his or her environment (e.g., addressing Lazarus's transactional stress) [4]. Of these approaches, the last one was the most appraised, as observed by Randall and Bodenmann [5].

In this way, Selye [6] described the general adaptation syndrome (G.A.S.), in which he suggested that the body reacts in a very similar way to a wide range of harmful stimuli. According to Selye [6], G.A.S. encompasses a vast array of response reactions of the body through which it defends itself against stressors (from a microbial infection to a strong emotion). G.A.S. comprises 3 phases: the “alarm reaction” stage, the “stage of resistance,” and the “stage of exhaustion.” The majority of processes happening during the AR stage, for example, a catabolic state, hypoglycemia, digestive wearing away, release of adrenaline, and hemoconcentration, revert to their prestress levels and functioning in the second stage. However, ongoing stress starts off the final stage, where the phenomena mentioned in the AR stage emerges back again [6]. During the alarm reaction stage, initially characterized by shock and decreased resistance to stressors (i.e., heightened senses and increased heart rate) is therefore designed to prepare the body to cope with stressors [3]. In the countershock phase, the biological defense reaction begins, manifested by increased respiratory rate, muscle tension, blood pressure, blood sugar levels, hormones, and cortisol in the bloodstream [3]. If the stimulus ceases, the body returns to its original balance [3, 6].

If its action continues, the body enters the stage of resistance, in which the body actively copes with the stressor. Defensive reactions intensify, and the acquired adapting process is maintained [3, 6]. If the stressor ceases, it returns to its original balance, but if it holds for too long, the body becomes depleted. The exhaustion stage is reached when the person has failed to adapt, when the subject can no longer defend himself. In this case, physical or mental illness sets in.

The stress defense mechanism is represented by processes that unite cognition and behavior and their physiology [7], acting together as a shield against the vicissitudes of the outside world, thus restoring homeostasis and ensuring survival of the species (Table 1).

The brain mediates the assessment and acknowledgment of environmental stressors but also other stimuli in order to establish and induce cardiac and vasculature adjustments, as well as adapt immunologic and endocrine processes [9]. Specific molecular alterations are made during these procedures, leading to changed neural pathways, efflux of chemical mediators, and the ultimate remodel of physiology [9]. Brain regions that play a part in the stress response encompass structures like the hippocampus, amygdala, and prefrontal cortex. The hypothalamus, which activates the sympathetic nervous system (SNS), leads to the release of adrenaline into the bloodstream, but also cortisol through the stimulation of ACTH hormone [10]. Also, the prefrontal cortex, the most evolved area of the brain, acts as a control center by handling emotional responses, thoughts, and actions. The brain stem, another key component, manages autonomic control but also how the body reacts to stressful stimuli. The amygdala, the so-called “brain's threat detector,” belongs to the limbic system and is known for managing emotions, processing threats, modulating fear responses and behavior, and consolidating memories [11]. Lastly, the striatum, found in the center of the brain, controls cognition and reward and coordinates movements [12]. Sudden distress triggered by a chain of events as well as prolonged nervous tension after being subjected to multiple or recurrent traumatic experiences precipitates the progression of psychological warfare and deteriorates interpersonal skills, subsequently engaging psychopathology [13].

**Figure 1:**
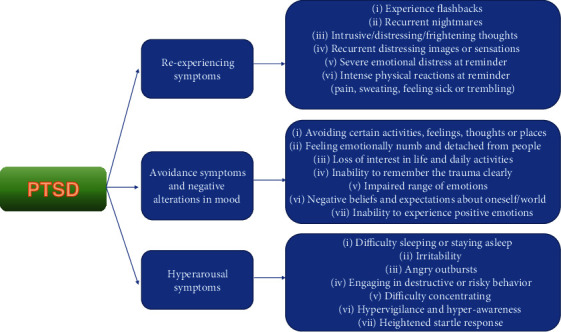
Symptoms of PTSD [16].

Regarding the frequency of this disorder across the world, research concerning epidemiology shows that roughly 3/4 of citizens will encounter a distressing experience, but solely 1/5 of the people who experienced major stress are prone to be diagnosed with posttraumatic stress disorder [14]. This syndrome has a wide range of manifestations, mostly parted in three categories: reexperiencing symptoms, avoidance symptoms, and hyperarousal features [15] (Figure 1).

The remarkable trait of the pathophysiology is that the fear response cannot be suppressed, and therefore, people with posttraumatic stress disorder relive distressing recollections, not being able to hold back the physiological reactions when dealing with traumatic impulses. Occurring modifications in neuroendocrine systems as well as modulation of metabolism, inflammation, or neurotransmission were illustrated in patients suffering from PTSD [17]. Furthermore, the stress disorder is linked to a range of different psychiatric conditions (for example, anxiety and panic disorders, depression disorder, impulse control disorders, addiction to various substances, legal/illegal drug abuse, and suicidal behavior) and somatic ailments and complaints (for example, heart and blood vessels, thyroid, pancreas, digestive, lung, muscular, and dermatological diseases) [18, 19].

## 2. Somatization Correlated with PTSD

Most of the time, those who have been through traumatic situations encounter different modifications related to physiology, onset or aggravation of somatic ailments, unexplained symptoms, somatization disorder, and other somatoform disorders [20, 21].

Somatic symptom disorder is depicted as an exaggerated concern over physical symptoms, such as weakness, pain, headaches, or tremor, which usually leads to emotional discomfort and troubles in daily life functioning [22].

Symptoms can vary from localized sensations, such as pain in the chest area or pain in joints, to generalized symptoms, such as dizziness, movement disorders, or weakness; they can also have no identifiable medical cause, or they can be related to a specific medical issue but with a higher intensity than expected [22].

Somatoform disorders are represented by physical symptoms that do not possess a medical explanation and unlike dissociative disorders; their onset is progressive, as the patient is constantly addressing the medical services for further examinations and investigations, despite not finding a somatic cause or justification [23].

Complaints can involve any part of the body, but most often, patients present with digestive ailments (abdominal distress, bloating, nausea, vomiting, diarrhea, and constipation) and also dermatological symptoms, such as local/generalized pruritus, urticaria, burning, and numbness [23]. Also, the somatization disorder is more commonly found in women, with its onset happening in their 20s. More so, there have been reported multiple severe situations which frequently side with impairments of personal, relational, and professional life.

Patients who suffer from posttraumatic stress disorder often presented with somatic afflictions which cannot be explained by organic pathology, like blurry vision, unexplained vertigo, tinnitus, and somatoform disorders [24]. They can also display a variety of medical conditions such as cardiac, lung, neurological, muscular, digestive, immunity, and gynecological disorders; generalized physical complaints; chronic pain; diabetes; and sleep disturbances. This pathology encompasses limbic instability as well as modifications in the HPA axis and sympathoadrenal medullary axis, which subsequently can alter neuroendocrine activity, as well as immunity, leading to autonomic nervous system dysregulation, pseudoneurological symptoms, and dysfunctions in sleep dynamics [24].

Even though a past filled with traumatic experiences is firmly essential to the existence of somatic symptoms, but not everyone who experiences distress in their lives is prone to psychological disturbances and somatization. A number of reasons for this variability involves diversity of genes, personality types, and demographical factors (for instance, more than 30% of soldiers diagnosed with posttraumatic stress disorder display serious physical symptoms, while the rest of them show no sign) [25]. Moreover, according to past observations, females displayed a predisposition towards developing somatic symptoms, such as nausea, tremors, pain, fainting, dizziness, headache, stomachache, and vomiting. Nevertheless, the intricate mechanism that connects trauma exposure to adverse mental health is only partially understood.

The link that connects terrifying experiences at a tender age with the development of somatic symptoms was revealed by researchers, including Heim and colleagues [26], who acknowledged a connection among abusive relations in early life and a physical ailment called “chronic fatigue syndrome.” In a similar fashion, Waldinger, together with his associates [27], realized that children who experience persistent trauma become more vulnerable and develop somatic symptoms, while they also form insecure attachments over time.

Chronic PTSD, especially complex PTSD, was linked to a high rate of autoimmune conditions, including Hashimoto's thyroiditis, type 1 diabetes, and Crohn's disease. It was Boscarino [19] who postulated that “biological mediators of these conditions might have a clinical correspondence with higher T cell counts, higher IgM levels, hyperreactive immune responses on delayed cutaneous hypersensitivity tests, and also lower dehydroepiandrosterone levels.”

Another frequent comorbidity among patients diagnosed with PTDS is cardiovascular disease [28]. A number of researchers illustrated that manifestations of posttraumatic stress disorder are connected to coronary heart problems. Chronic sympathetic arousal emerging from anxiety is crucial for the progression of cardiac disease employing reduced heart rate variability [28]. Other points of order lean towards high action of the SNS and low one from the PNS, universally known for causing the most perilous abnormal heart rhythms: ventricular fibrillation and ventricular tachycardia and ultimately cardiac arrest. Stress disorder presents a higher vulnerability for the development of cardiac disease, identifying a faulty pattern of AV transmission and also necrosis on the electrocardiography. On the other hand, depressive disorder is linked to surfacing arrhythmias [29].

## 3. The Interrelation between Gut Microbiome and Other PTSD-Related Distresses of the Gastrointestinal Tract

The individual microbiome has recently come under the spotlight for its potential contribution to individual variability in risk of developing posttraumatic stress disorder after having been exposed to stress due to its various interactions with the host, including effects on neural, neuroendocrine, and immune signaling [30]. The gut microbiome has been hypothesized to influence the cerebrum and cerebral activity in a matter of managing neurotransmission, generating compounds which regulate immunity functions brain and also toxic substances released by bacteria.

The intestinal microbiome consists of all microorganisms and their genes located in the gut, while “gut microbiota” refers solely to the living microorganisms (archaea, bacteria, viruses, and eukaryotes, such as fungi) [31]. The compositions of individual microbiomes emerge through an intricate interplay between genetics and environment, with the latter playing a dominant role into the story. The dynamism of the microbiome is defined by its ongoing change depending on lifestyle factors.

It is specified in the literature that at least one thousand organisms of the bacterial biomass colonize the human gut with at least 160 different species per individual, and cumulatively, these bacteria store an estimate of 150 times more genetic information than the human host [32].

Where digestion occurs, the bacterial phyla *Firmicutes* and *Bacteroidetes* encompass around 3/4 of the intestinal population, the two species being particularly sensitive to change [33]. Alterations of the microbiome are becoming more and more linked to the vulnerability towards allergies, disorders that target the immune system, diabetes, and psychiatric and neurological ailments, which have a widespread presence among people from all around the world.

As stated before, stress, emotions, and trauma have the power to modify the bacterial composition in the digestive tract. Cortisol and epinephrine interfere with the proliferation of bacteria, causing harm to the gut mucosa and therefore leading to an outflow of toxins and bacteria into the bloodstream. This process gives rise to inflammation, an etiology factor incriminated in various psychiatric disorders [33]. Research shows that gut bacterial biomass takes a toll on brain functions, thought processes, the ability to memorize, and also behavioral actions, sociability, and coping with emotional tension [31].

Knowledge is that the central nervous system controls gastrointestinal function in the gut, but it is partly clear how the gut habitat, including the microbiota, may influence brain function, especially in the realm of psychiatric disorders, such as anxiety and depression [31, 33]. Brain-gut-microbiome signaling pathways, including efferent neural, neuroendocrine, and immune pathways engaged by the central nervous system, work side by side to mediate homeostatic responses in the gut [30].

The possibility to develop PTSD as well as the symptom constancy may be influenced by the bidirectional signaling of the microbiome-gut-brain (MGB) axis. Alterations in microbiota proved to “modulate plasticity-related, serotonergic, and GABAergic signaling systems in the central nervous system” [32].

The gut-brain axis (GBA) refers to dialogues happening both ways amid the central nervous system and the digestive tract (the enteric nervous system), with the purpose of monitoring and integrating intestinal activities and also engaging the cerebral areas responsible for cognition and emotions and linking them to the gut features, namely, intestinal permeability, “enteric reflex,” immune activation, and enteroendocrine signaling [33].

**Figure 2:**
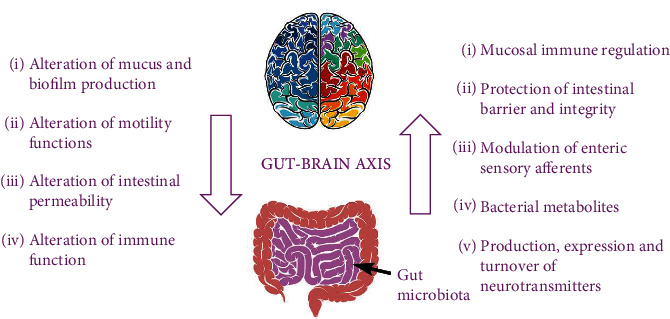
The gut-brain axis and principal mechanisms of bidirectional communication that happens amid the central and the enteric nervous system [33, 34].

This complex communication network, known as the GBA, comprises a vast number of mechanisms and elements such as the hypothalamic-pituitary-adrenal (HPA) axis, which is a fundamental component in how the body responds to stressful stimuli, immune cells, such as cytokines and chemokines, the vagus nerve, which is primarily involved in the mind-body connection, short-chain fatty acids (butyrate), neurotransmitters, and neuropeptides like serotonin (5-HT), GABA, dopamine, leptin, melatonin, histamine, and acetylcholine [34]. With tryptophan being the major serotonin precursor, interference with its metabolism represents a key factor, as more than 90% of serotonin is released by enterochromaffin cells found in the gastrointestinal mucosa. Serotonin plays an essential role in the brain, where it modulates mood, cognition, anxiety, and the learning process, as well as in the gut, impacting bowel function by modulating secretion, influencing motility and pain perception. Other components of the GBA are represented by gut permeability, which ensures the transportation, absorption, and balance of nutrients and also immunity, gut microbiome, and autonomic nervous system (ANS), engaging both afferent (sensory) signals, modulating gut motility and pain perception through calcium-dependent potassium channels and also efferent (motor) signals [33, 34] (Figure 2).

The GBA relies on reciprocity, as the central nervous system influences the processes occurring in the digestive system through motility, secretion, nutrient delivery, and microbial balance, while the gut also modulates how the brain operates, impacting mood, behavior, stress, and anxiety. Microbiota interacts with GBA through various mechanisms, primarily by regulating the intestinal barrier, which, if altered, influences and affects all the fundamental compartments.

Dr. Stefanie Malan-Muller conducted a study where she analyzed the gut microbiomes that belonged to individuals suffering from posttraumatic stress disorder. Afterwards, she gathered the same samples from people who have also had some tough life experiences, without developing the disorder (trauma-exposed controls) [31]. The team compared the two lots as they found a preponderance of 3 bacterial species, namely, *Actinobacteria*, *Lentisphaerae*, and *Verrucomicrobia*, with significant differences in the PTSD lot (scarce number of the aforementioned species) in comparison to the other lot [31]. However, a reduced number of *Actinobacteria* and *Verrucomicrobia* were found in samples taken from people who faced adversities in their early years [31]. It is noteworthy to say that adults who faced childhood adversities are more likely to develop this disorder at some time in the future, having already set in motion a chain of events happening at the site of the microbiome in response to what they have previously experienced.

Furthermore, scientists have revealed how early-life stressors can alter the gut microbiome [35]. Emotional tension and HPA axis dysfunction can affect or induce a condition called SIBO (small intestinal bacterial overgrowth) through several mechanisms, such as a scarcer gastric acid secretion, a slower digestive motility, altered level of secretory immunoglobulins, a bigger vulnerability towards pathogens, an increased virulence, and the accumulation of biofilm [36].

SIBO is often deemed responsible for poor absorption of food nutrients, as well as chronic diarrhea, being characterized by the invasion of colonic bacteria proximally into the ileum and jejunum, with the bacterial population exceeding 10 [5]–10^6^ organisms/mL (the standard multiplied by a thousand) [37–40]. By competing for fundamental nourishment, the bacterial flora is likely to alter the host's metabolism, impacting the mucosal lining of the individual, as well as generating digestive phenomena that lower/change dietary consumption [38].

Poorly absorbed nutrients (malabsorption) are a typical feature of this condition, with the possibility to lead to different ailments in the individual. Individuals who suffer from bacterial overgrowth could equally experience unexplained slimming, as well as vitamin deficiency (B_12_—leading to anemia, vitamin D—causing osteoporosis, and vitamin A—vision impairment). This condition is named blind loop syndrome [37].

SIBO generally occurs due to faulty or abnormal behavior in systems which are trying to maintain homeostasis by controlling gut microorganism colonies. Reduced secretion of gastric acid and dysmotility in the small intestine are often associated with predisposition to bacteria overcrowding [37], as well as abnormalities in the anatomy of the GI tract and immunity impairment, which can amplify the risk for bacterial overgrowth. In time, their accumulation can result in inflammation of the gut mucous membrane, which ultimately aggravates the typical symptoms of SIBO [37].

The prevalent symptoms include pain or discomfort in the abdomen, diarrhea, fatigue, meteorism, weakness, and excessive gas [41]. Several patients only mention one or two complaints; they can either present with vitamin deficits or just a few dropped pounds. Others experience explosive diarrhea or fatty stools. Symptom frequency and severity directly correlate to the level of overpopulation, as well as the gastric mucosal degree of impairment [37].

The mechanism through which the risk of developing SIBO increases holds accountable an impaired migrating motor complex (MMC) [36]. By prompting food to linger in the small intestine, it provides a favorable and nurturing environment for bacterial populations to increase abnormally. The MMC is a periodic coordinated movement of myoelectrical waves, which uses contractions of the GI smooth muscles during fasting and has a glaring effect on the peristalsis through the GI tract [42]. It is known to play a “decluttering” part by moving undigested residue through and out of the GI tract [42]. Evidence suggests that stressful situations act as direct inhibitors for MMC [43]. Beaumont postulated that anger, fear, or any emotions whatsoever that devitalize or disrupt the nervous system were linked to suppression of digestive motility and altered digestive function [44]. It is known that corticotropin-releasing factor (CRF), a fundamental regulator of the HPA axis, mediates the suppression of the MMC (in times of nervous tension) [36]. Upon its release by the hypothalamus, corticotropin-releasing factor will bind to brain receptors, impairing the synaptic transmission governing the migrating motor complex [45].

Stress is yet another cause for gastrointestinal motility dysfunction, as it generates significant fluctuations in blood sugar levels [36]. Elevated levels of cortisol on account of prolonged stress produce glycemic fluctuations. These spikes and drops therefore encourage prolonged periods of hunger, resulting in more frequent meals (ergo increased food intake), ultimately reducing the wait time from one meal to the next, which is when the migrating motor complex is the busiest, therefore negatively impacting GI kinetics [36].

Moreover, stress response mediators, such as cortisol and catecholamines, set the ground for the aforementioned biofilm formation (represented by a population of microorganisms that share common DNA and nutrients, adapting skills such as eluding the immune system, acting like a shield against antimicrobial treatments, resulting in infections that are hard to cure), by aiding harmful bacteria retrieve nourishment as a means to survive [46–48].

Regarding the gastrointestinal distresses, another pathology comes to light when it comes to its connection with PTSD. IBS recognizes the existence of predisposing or aggravating factors such as psychosocial stressors in its onset [49]. Unlike patients with structural gastrointestinal disorders, FGID patients are more likely to have suffered emotional disturbance or to have experienced life-threating situations [49].

IBS or irritable bowel syndrome represents a functional bowel disorder, with its main features being recurring abdominal pain happening weekly for a three-month period and that is associated with two or more of the following: differences in the defecation process, a modification in stool appearance or a perceived difference in bowel habits (frequency) [50]. Around 1/5 of adult population as well as teenagers universally experience these symptoms and more, predominantly in women, as indicated by research [51, 52]. Patients usually experience an array of comorbidities such as meteorism, cramping, constipation/diarrhea or both, and bowel incontinence, but also emotional and social distress [50]. Irritable bowel syndrome has been linked to conditions such as anxiety, depression, and a stressful living situation, while considering the GBA indispensable for understanding IBS [53].

The intricate matrix of etiologies likely constitutes a variety of circumstances targeting pathophysiology, which may vary from one individual to another, including “visceral hyperalgesia,” “leaky gut (intestinal permeability),” activation of the immune system, GI motility modifications, “autoimmunity,” and alterations of intestinal microbiota [54].

As we previously stated, patients suffering from IBS are likely to be more vulnerable and susceptible to emotional and psychological turmoil, presenting a higher rate of comorbidities among mental disorders, including anxiousness and stress, but also low dynamism/vitality, poor quality of sleeping patterns, and performance difficulties on a daily basis [55]. That is why many treatments target neurobehavioral intervention and tackling the use of antidepressants.

A number of hypotheses postulated a potential connection between irritable bowel syndrome and PTSD. IBS has a multifarious pathogenesis, having psychological, social, hereditary, endocrine, central and enteric nervous system, visceral sensitivity, and hyperalgesia as well as infectious and/or inflammatory elements [56]. Tanaka et al.'s “biopsychosocial model” for the aforementioned disorder promotes this conceptualization [57]. Therefore, the circumstances influencing the onset and progression of IBS point to genetic factors and “social learning” as belonging to childhood and formative years, psychological and social events (mistreatment, emotional upheaval, stress, and mental frame of mind), and pathophysiological factors, such as alterations in terms of sensitivity and motility—stronger and longer contractions lead to meteorism and diarrhea—and also dysregulation of the HPA axis. Another key component is the communication between the brain and the digestive system, where defectively coordinated signals can trigger pain or bowel movement modifications. Even more, irritable bowel syndrome has the possibility to develop after a bacterial or viral infection of the digestive tract [54].

For instance, a study conducted by Ringel and his team [58] utilized PET scans in order to observe cerebrovascular perfusion in people suffering from IBS in comparison to the control group, along with subsequent examinations and interpretations, differentiating individuals who presented a background of sexual assault or physical mistreatment as opposed to people showing no signs of abuse. The results showed a higher activity spike in the “cortex cingularis anterior” for individuals without irritable bowel disorder and for the people without a background of abuse. IBS patients were linked to greater thalamic activity, this region being responsible for nociception response.

Going back to the topic at hand, concerning dysbiosis, a number of studies have shown promising results. For example, in a mouse model displaying distressing pressure in early stages, mother estrangement results in microbial dysbacteriosis, enhanced HPA axis activation, and increased anxious reactions and protective/aggressive behavior later on [35]. Another study showed how maternal stress during pregnancy leads to increased concentrations of glucocorticoids (cortisol) determined in the saliva. This proved to actively impact the infant's microflora configuration and diversity, as maternal stress consequently altered the infant's microbiome throughout their first 4 months of life [59].

It is well established that people with mental health problems caused by stress, like anxiety and depressive disorder, exhibit altered microbiota abundance and higher gut permeability [60]. Animal models who experienced social defeat (ongoing stress) presented with a remarkable decrease in terms of relative abundance and general diversity of many bacterial species (such as *Akkermansia* spp.), as well as less frequent signal transduction pathways, such as seven-transmembrane domain receptors that undoubtedly correspond to quantitative assessment criteria found in major depressive disorder [60]. Moreover, traumatic physical stressors are linked to major alterations in the gut flora, where the changes take place within 72 hours of the injury, leading ultimately to dysbiosis, higher gut permeability, and an elevated concentration of circulating proinflammatory cytokines [32]. Furthermore, activation of the HPA axis will lead to increased secretion of glucocorticoids, ultimately causing dysbiosis as well.

Another sine qua non element is the sympathetic nervous system, which, if stimulated for an extended period of time, can change microbial composition in the gut and increase gut permeability, caused by the increase of catecholamines released by the adrenal gland and sympathetic nerve terminals [61]. It was also demonstrated that epinephrine and norepinephrine could induce the proliferation of a variety of gram-negative bacteria, especially *Escherichia coli* [62]. These bacteria also proliferate after injury-induced release of norepinephrine, where the SNS is involved as fundamental player in intestinal colonization of gram-negative bacteria and gut dysbiosis [63].

As presented before, emotional upheaval is a fundamental trigger that influences the alteration of digestive microflora, as well as the intestinal mucosa barrier function. Changes to the intestinal microbiome on account of stress during childhood, a susceptible time when the digestive microflora configures the homeostatic immunity functions and the neurological pathways of the host, may carry deep-rooted repercussions regarding immunity, spiking the likelihood for subsequent illnesses related to stress in the future and contributing to a proinflammatory state and low cortisol in adulthood [32].

## 4. The Key Part Inflammation and Oxidative Stress Play in PTSD

Recent evidence has come forth suggesting that some PTSD outcomes combine high systemic degrees of oxidative stress (OXS) with inflammatory activation. Inadequate regulation of the immune system and increased inflammatory levels were found to be potential risk factors, while bacterial resources play a major part in immunoregulation [64]. On the other hand, complex posttraumatic stress disorder is a type of recurrent and long-term trauma which increases oxidative stress and puts cell senescence into overdrive [65]. Where inflammation (INF) occurs, different biological catalysts are released, including reactive species of oxygen and nitrogen, proinflammatory cytokines, and other molecules, thus inducing oxidative stress. Oxidation and inflammation therefore have the tendency to happen at the same time, as both of these processes are likely to cause the onset of the other.

Inflammation is how the immune system responds from a physiological point of view to a variety of factors and injurious situations, including infectious agents, cellular injury, harmful substances, or irradiation [66]; the immune system reacts by fighting against pathogens, followed by elimination of cellular debris and ultimately launching of the recovering process [67]. INF is, ultimately, a crucial biological warrior and the key to survival and adaptation [68].

Triggers can produce acute or chronic inflammation in cardiovascular, hepatic, renal, pancreatic, respiratory, cerebral, digestive, and genital systems, having the potential to trigger tissular injuries and disease [69]. Inflammatory cells become activated by (non-)infectious agents, as well as cellular injury which eventually sets off inflammatory signaling pathways. As a reaction to tissue injury, the body fights back by initiating a chemical signaling cascade which will therefore stimulate responses in order to heal the damaged biological material. The signals trigger and attract leukocytes through chemotaxis, directing them from the bloodstream to the injured sites. After their activation process, leukocytes secrete cytokines, which regulate inflammation and also act as immunomodulating agents [70].

The action of different biological catalysts such as reactive species of oxygen and nitrogen, proinflammatory cytokines, and other mediating compounds leads to oxidation. Therefore, inflammation and oxidation as processes have the tendency to happen simultaneously, also being capable of inducing one another [65]. Altogether, these concepts can be triggered and severely impacted by psychological stress, especially when it occurs at length.

Regarding the neuroinflammatory component in the pathophysiology of PTSD, evidence suggests that considerably high levels of mRNA as well as specific mechanisms that produce cytokines were found in the brain [71]. Even more, the potency of oxidative stress extends past the brain, involving the adrenal glands, as well as blood, proving that PTSD has the potential to develop into a systemic condition which involves multiple organ systems. Up-to-date studies have shed light on amplified inflammation processes found in the pathophysiology of posttraumatic stress disorder; for example, it was found that T regulatory cells (Treg) were modified in stress-afflicted patients. Treg play a key part in defending the body against inadequate inflammation effects, such as those encountered in autoimmune disorders, allergies, and asthma [72].

Furthermore, decreased amounts of Treg were found following the human participants' in-lab stressor subjection. To this point, reduced T regulatory cell frequency is linked to autoimmunity phenomena found in disorders like Hashimoto's disease, IBD, and rheumatoid arthritis, as people diagnosed with posttraumatic stress disorder presented an elevated risk for such conditions [72, 73].

In line with these discoveries, genome-wide association studies (which identify which genes are responsible for certain pathologies) on groups of people who suffered from posttraumatic stress disorder discovered an important connection with the Ankyrin Repeat Domain-55 gene, which is linked to a series of inflammatory and autoimmune diseases, such as MS, T2D mellitus, gluten-sensitive enteropathy, and rheumatoid arthritis [74].

Furthermore, posttraumatic stress disorder was found to cause upregulation of serum interleukin 6 (IL-6) and proinflammatory cytokines, such as interferon-gamma (IFN-*γ*), interleukin 1 *β* (IL-1*β*), interleukin 10 (IL 10), and tumor necrosis factor *α* (TNF *α*) [75–77]. Studies have shown that proinflammatory cytokines increase in concentration post exposure to stress, but also when dealt with secondary symptoms of stress, such as fatigue and sleep disturbances. High concentrations of CRP (C-reactive protein), an inflammation marker used clinically, were also found in individuals with PTSD, correlating with symptom severity [75, 78].

Glucocorticoid-mediated immunosuppression can lead to the short-term decrease of inflammation, but eventually may also trigger a homeostatic inequity among the mucosa, digestive microflora, and pathobionts (potentially pathological microorganisms). Cortisol does cause a boost in commensal pathogens like Helicobacter pylori, bacteria known to amplify local and systemic inflammation and lead to chronic ailments like gastritis [79, 80].

In an experiment, animal models subjected to negative psychological stimuli display an increase in Helicobacter species, assessed via RT-PCR to determine absolute/relative abundance; in order to prevent this effect, it was recommended to administer an antiglucocorticoid [81, 82]. Helicobacter spp. proved to cause inflammation in the inner lining of the colon in IL-10−/− rodents, with inadequate regulation of the immune system, a potential effect of the exaggerated immune defenses of the host [83]. The inoculation process using a tyndallized preparation based on bacteria with immune-regulating properties that boosts Treg and anti-inflammatory cytokine levels proved to avert stress-induced spikes in a disease similar to posttraumatic stress disorder in rodents, which would suggest that tipping the scales of pro- and anti-inflammatory elements might play a significant role in developing a PTSD-like syndrome [81–83].

Oxidative stress is a phenomenon attributable to a disturbance in homeostatic balance between pro- and antioxidants resulting in the production of reactive oxygen species (ROS), which have predominantly beneficial effects to cells, except for the time when they exceed their basal level. Certain mechanisms that promote oxidation will trigger molecular signaling pathways, consequently generating free radicals, as well as reactive nitrogen species (RNS) [84].

**Figure 3:**
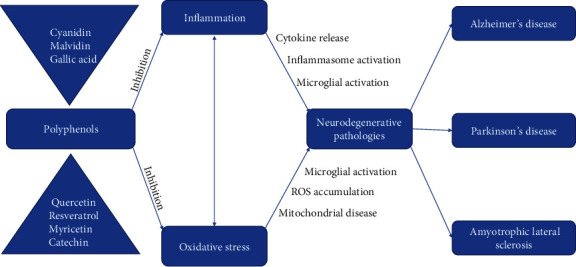
Polyphenol inhibition of neurodegenerative mechanisms [91].

OXS happens when levels of ROS (superoxide anion, hydrogen peroxide, hydroxyl radical, peroxyl, nitric oxide, and reactive aldehyde or other prooxidant molecules) are so high that the present antioxidant molecules (superoxide dismutase, glutathione) cannot counterbalance the repercussions. Reactive oxygen species cause harm by oxidizing proteins/enzymes, carbohydrates, and lipids, as well as DNA or RNA and other cell elements, leading to functional cellular alterations, tissue necrosis, and upregulation of proinflammatory cytokines. Oxidized proteins will present themselves with the prospect of substantial cytotoxic consequences. When it comes to the homeostasis of cells, there are certain physiological fluctuations regarding the cellular redox state; however, an increased level of oxidative stress will lead to pathological modifications of cell signaling, thus gifting the cell with an inflammatory phenotype. OXS is an elementary biochemical senescence process, as the capacity of the body to counterbalance it is crucial for ensuring somatic welfare, lastingness, vitality, and endurance [85]. Furthermore, oxidation sets off signaling pathways that promote inflammation (and the other way around) [86, 87] and is involved in the etiopathogenesis of several health problems, like cardiovascular disease, chronic obstructive pulmonary disease, cancer, diabetes, and neuropsychiatric or neurodegenerative conditions such as psychotic disorder, Alzheimer disease, amyotrophic lateral sclerosis, Parkinson's disease, multiple sclerosis, autistic spectrum disorder, and bipolar disorder [88–91] (Figure 3).

Inflammation and oxidative stress represent key parts inside the pathogenesis of neurodegenerative disorders, as well as psychiatric ones. Research shows that phytochemicals such as polyphenols (flavones, flavonols, flavanones, flavanols, stilbenoids, anthocyanins, etc.) that are usually found in compounds such as cocoa, olive oil, grapes, berries, coffee, tea, and peanuts could exert a positive effect on the intrinsic mechanisms of these disorders on account of their antioxidative effects [91].

Recent evidence shows that psychological disorders that develop after exposure to stress, such as posttraumatic stress disorder, as well as persistent emotional tension, can trigger and influence the concept of oxidation and inflammation, which means that their chronic effects and exposure have destructive consequences on the brain [92].

Of all structures, the cerebrum harbors the biggest vulnerability and susceptibility to the effects of oxidative stress due to its elevated metabolic demand, increased use of carbohydrates and O_2_, and dense composition of oxidation-prone lipids [93]. In the central nervous system, the consequences of OXS are represented by a higher blood-brain barrier permeability, difficulties in neurotransmission, defective synaptic plasticity, disruption of neurogenesis, altered patterns of neural growth, and remodeling of neural morphology. It is also well established that OXS takes a toll on neural cell destruction and lesions in neurological conditions such as Parkinson's and Alzheimer's disease [94].

High levels of oxidation stress serum biomarkers were also found in healthcare workers who have experience prolonged, intensive nervous tension, with significant degrees of apprehended tension linked to increased extent of nucleic acid and lipid oxidative injuries [95]. Research proved that in overburdened people, the connection among the stress they felt and the injuries oxidative stress inflicted was mediated by cortisol [95]. Similarly, inquiries conducted on individuals manifesting anxious behavior revealed increased degrees of lipid peroxidation in subjects with generalized anxiety disorder [96] and inhibited antioxidative activity in subjects with panic disorder [93]. There have also been studies that suggested that individuals diagnosed with depression display inhibited antioxidative activity and high extent of nucleic acid oxidative injuries [97].

Several animal models involving rats postulated that a prolonged exposure to stress leads to increased ROS levels in certain regions of the brain, namely, the prefrontal cortex and the hippocampus. Scientists also observed features such as weight gain, a higher body temperature, and changes in the liver and heart histopathology depicting tissues with inflammation and fibrosis [98]. They also found a significant level of OXS-related proteins in plasma, as well as a high inflammation state.

When it comes to ailments of the gastrointestinal tract, the mucosa acts as a protective barrier, which can be overpowered by microbial pathogens and ingested compounds, triggering inflammatory responses, but also oxidative injury at the site. Disorders such as peptic ulcers, neoplastic diseases of the gastrointestinal tract, or inflammatory bowel disease have pointed to oxidative stress as an underlying mechanism of the pathogenesis [99].

As previously discussed, ROS are produced within the gastrointestinal tract and they serve as indispensable signaling molecules. For example, they are mainly used by cancer therapy, such as chemotherapy or radiotherapy, in order to induce apoptosis and ultimately remove malignant cells. However, the body homeostasis can be easily disturbed when there exists a disproportion of ROS generation, as they are largely fabricated with regard to agents such as cigar smoke, alcoholic beverage intake, administration of NSAIDs, or processes such as infections or ischemia-reperfusion (I/R) injury [100].

Inside the GI tract, ROS are predominantly generated by two enzymatic reactions: the hypoxanthine/xanthine oxidase system and the NADPH oxidase system. Xanthine oxidase turns hypoxanthine in xanthine and, later on, to uric acid (both reactions generating O_2_^−^) [100]. In the ischemia process, there is an increased production of xanthine and xanthine oxidase, at the expense of antioxidant enzymes, therefore generating ROS species such as O_2_^−^ and H_2_O_2_, leading to an impaired digestive system [100]. A disruption in the GI tract barrier will lead to an altered gut permeability, ultimately contributing to inflammation by stimulating PMNs.

Moreover, emerging studies suggest that neurological degeneration related to posttraumatic stress disorder might have something to do with the condition's extent and intensity, meaning that the neuronal repercussions are directly proportional to the period during which a patient experiences the aforementioned disorder [93]. Phenomena such as flashbacks and intrusions are associated with activation of neuronal circuits related to fright, an amplified catecholamine and cortisol output, and also an increased peripheral autonomic nervous system activity.

Research based on structural brain imaging discovered clear correlations amid posttraumatic stress disorder and shortfalls of neuronal wholeness in places such as the amygdala, hippocampal region, and the medial prefrontal cortex, as well as the anterior cingulate cortex [94].

The hypothalamic-pituitary-adrenal axis is a fundamental physiological pathway, ruling the stress response system. Alterations of its neuroendocrine functions proved to be associated with the pathophysiology of PTSD [101], and its long-term incessant stimulation impacted negatively the cerebral structures.

“The glucocorticoid-hippocampal atrophy model” [102] states that stress-related secretion of glucocorticoids (GC) leads to a degree of neurotoxicity at the CNS level. Because of its abundance regarding GC receptors, the hippocampus is explicitly at ease. A variety of research performed on animals revealed that high concentrations of GC are linked to an elevated number of reactive oxygen species as well as injuries resulted from oxidation.

Another common symptom of PTSD is represented by sleep disturbance, on which OXS has a resounding effect if we take a look at what lies beneath. The sleeping process is fundamentally important for the cerebral structures and mechanisms when it comes to detoxification and restoration. This can manifest as recurring night terrors, disruptive sleeping patterns, and dyssomnia or parasomnia [103, 104].

While sleeping, neuronal labor is diminished, thus favoring antioxidative processes [105]. This represents an essential process considering how sleep, through its five stages, restores the mind and body, as revealed by drops in antioxidant agents and remarkable spikes in oxidative biological markers following lab-induced sleep deprivation [106, 107]. Similarly, sleep deprivation has led to higher proinflammatory molecule concentrations (TNF*α*, IL-6), as well as C-reactive protein. As it is known, the sleeping process is crucial for sustaining memory and cognitive functions, as well as learning, cerebral detoxification, and encouraging neuronal rehabilitation [105, 108]. Extended wakefulness periods lead to a cerebral abundance of reactive oxygen species because of increased conversion of oxygen into energy [109]. There have also been animal studies that support the hypothesis according to which regular poor sleep triggers oxidative stress in the hippocampal area, thus leading to shortcomings when it comes to memorizing. Therefore, by using antioxidants, this effect can be counterbalanced [110].

To sum up, evidence from across a variety of studies (clinical, neuroimagistics, etc.) supported the premise that complex posttraumatic stress disorder is correlated with increased levels of oxidative stress [65]. Moreover, oxidative stress together with inflammation develops progressively alongside the aforementioned disorder, thus providing a modern perspective over therapeutic interventions.

## 5. Metabolic Disorders and Their Connection to PTSD

Clinical data backs up the hypothesis according to which beneath the impairment of fright suppression functions, posttraumatic stress disorder also represents a metabolic disorder on the basis of modified function of inflammatory response mechanism, involving neurological pathways like the hypothalamic-pituitary-adrenal axis, SNS, and inflammation [17].

The metabolic syndrome (syndrome X) is characterized by the succeeding clinical features: excessive body fat around the waist (central obesity), high fasting blood glucose (>100 mg/dL), hypertension (>130/85 mmHg), high triglyceride concentrations (>150 mg/dL), and low HDL cholesterol concentration (<40 mg/dL) [111]. Syndrome X as well as an increased body mass possesses a low responsiveness to leptin (a hormone released from the adipose cells), which leads to elevated plasma leptin concentrations and leptin insensitivity [112]. Before receiving its new name, this disorder was once called “insulin resistance syndrome” on the account of how important was this characteristic. An elevated blood sugar level happens in parallel with insulin resistance, as cells fail to react in a proper manner to insulin, thus leading to hyperglycemia [113]. Research has shown that the posttraumatic stress disorder can tangle with obesity and accompanying metabolic dysfunction [114].

The frequent comorbidity that occurs among syndrome X, type 2 diabetes, obesity, and posttraumatic stress disorder implies there are intrinsic neurological and endocrine alterations, as well as changes in the metabolism regarding the psychiatric disorder which can heighten the likeliness of a “systemic metabolic dysregulation.” It can also reveal a fundamental metabolic shift due to a stressful history [17]. Processes such as oxidative stress, heightened autonomic activity, glucocorticoid activation, or immunological dysregulation have been incriminated to lie at the foundation of this connection between PTSD and metabolic syndrome. Moreover, changes regarding pathways in inflammation subsequent to modifications in GC receptor responsiveness (secondary to emotional and physiological arousal) could represent the underlying basis for inappropriate social conduct concordant with posttraumatic stress disorder and the pathophysiological display of syndrome X [17].

A number of changes of the hypothalamic-pituitary-adrenal axis found patients suffering from posttraumatic stress disorder revert to “HPA axis-centric” modifications seen in dysmetabolic disorder, namely, excess body fat in the abdomen area [115]. As established before, the increased girth typical of syndrome X is linked to pathway modifications of the hypothalamic-pituitary-adrenal axis. As cortisol suppression is lowered [116], its reaction towards distressing stimuli is increased [117], as well as increased glucocorticoid levels observed at 7 a.m. [118, 119]. Thus, the heightened glucocorticoid activity and concentration are linked to hyperglycemia, insulin resistance, and high TG levels, key aspects for metabolic syndrome [120]. This being said, it suggests that the metabolic consequences resulting from the central stress response system joined by persistent emotional tension work synergically as a way of amplifying insulin resistance together with hyperglycemia formerly existing in metabolic syndromes [113].

The perceived modifications of the hypothalamic- pituitary-adrenal axis within the posttraumatic stress disorder are concurring with the activation of the SNS, therefore raising blood pressure and heart rate after subsequent exposure to stimuli of an acute stressor, thus acting as a predictor for the upcoming development of the illness [121].

Metabolic disorders are equally correlated with a heightened SNS activity [122, 123], such as enhanced muscle sympathetic nerve activity. Behavioral factors linked to PTSD such as unhealthy diet and insufficient exercise are likely to contribute directly to metabolic syndrome. There is a possibility that psychological symptoms and cardiometabolic processes exert epigenetic modifications expressed in the brain. Signs and symptoms of metabolic disorders and PTSD might impact the sturdiness of the interface that mediates the interaction between blood and cortex by increasing its permeability, thus enabling the dissemination of inflammatory compounds. Processes such as INF within the nervous system as well as OXS will eventually lead to decline in cognition and neural functioning [124].

Additionally, a high leptin level was discovered in the biological profile of people who experienced trauma, as well as in individuals who suffer from obesity [125]. Another common aspect between metabolic disorder and PTSD is insulin resistance, as the latter is correlated with a slight elevation of insulin concentration, as well as higher insulin responsiveness after performing a glucose tolerance test [126]. Similarly, data shows that the peripheral metabolism in PTSD is disrupted as well as the central glucose metabolism [17].

Overall, as certain baseline features seen in the metabolic disorder can be noticed in the pathophysiology of posttraumatic stress disorder, this further highlights the interconnection amid the aforementioned pathologies. Viewing the duality of posttraumatic stress disorder, further medical care can be directed towards the common links, in order to help individuals reach physical wellbeing.

## 6. Hematopoiesis Disbalance and PTSD-Related Manifestations

As stated before, psychiatric disorders in many instances present themselves with an imbalance regarding the bacterial population within the digestive system. A state of dysbiosis has been linked to obesity, irritable bowel syndrome, and even autoimmune diseases. Lately, evidence has shown that the gut microbial population influences altogether the process of generating blood cells inside the human body. Therefore, hematological alterations and gut dysbacteriosis have been associated with different health problems, namely, inflammatory bowel disease or metabolic disorders [127]. Furthermore, dysfunctions involving the production of erythrocytes, lymphocytes, and myelocytes have recently been shown in the posttraumatic stress disorder, while also linking them to the inflammation process.

The formation of blood cells is influenced by both external components, namely, cytokines and proteins that promote cell growth, as well as internal factors, such as genetic and transcription service, coordinating the differentiation of stem cells [128].

In a study conducted on mice for a period longer than two weeks, scientists observed that a therapeutic intervention using broad-spectrum antibiotics depleted and altered the gut microbial flora, influencing altogether the number of stem cells and their descendants found in the medullary cavities of the bones. Ultimately, this leads to anemia, pan-lymphopenia, and leukopenia due to the reduction and modification of gut microbiota caused by antibiotic treatment [100]. However, these effects can be easily reversed using the technique of fecal microbiota transplantation.

Recent studies showed the critical role of lipopolysaccharides (microbial components) that uphold the production of neutrophils and expression of Toll-like receptor/MyD88-mediated signaling [99].

The commensal gut microbiota supervises the adequate immune performance and hematopoiesis by involving microbial elements such as lipopolysaccharides to assist a constant production of neutrophils that fight pathogens using Toll-like receptor/MyD88-mediated signaling [99].

Even more, studies revealed that alterations of the microbiota are responsible for how well the body responds to different oncological interventions, such as chemotherapy and immunotherapy [129].

In another study conducted by Josefsdottir et al., the team postulated that commensal gut microbes are involved in regulating and sustaining normal hematopoiesis [100]. The gut microbiota manages the migration and phenotype while also influencing how numerous innate and adaptive cells behave.

Microbes residing in the gut prompt macrophages, dendritic cells, and also lymphocytes from the mucosa, which, therefore, trigger multiple extrinsic stimuli. These cellular and microbial stimuli sustain the tonic activity of hematopoietic progenitor and stem cells, together with white blood cells, such as neutrophils [99]. This will ultimately boost hematogenesis and provide the necessary tools for the immune system, with both its specific and nonspecific branches, to fight pathogens and limit the spread of the disease.

Alteration of bacterial communities in the gut sets the ground for increased susceptibility to a series of disorders [130]. Treatments, such as systemic antibiotics, can modify the diversity and shift the bacterial balance within the microbiome, leading to a compromised hematopoiesis and a higher propensity for infections.

In addition to influencing the body's defense mechanism, the microflora is required in order to keep systemic communities of neutrophils in the blood circulation and CD4+ T cells in the spleen, implying that the microbial population residing in the digestive tract plays a part in the ontogenesis of the immunity biosecurity [131].

## 7. Oxytocin and Cortisol and Their Influence in PTSD

Oxytocin, a peptide hormone and a neurotransmitter, may likely be an instrument that links childhood emotional upheaval with dissociative disorders, somatic symptom disorders, and PTSD [132]. Adverse events from a tender age, such as maternal deprivation, might leave their mark on how well those people react to stress with its neurobiological process, correlating persistent high amounts of plasmatic glucocorticoids and an enhancement of their sensitivity and receptivity to a various array of stressors, thus involving cortisol in this pathology [133, 134].

Oxytocin is a hypothalamic nonapeptide, secreted by the pituitary gland, which, as both a neurotransmitter and paracrine hormone, plays a vital role while engaging in a variety of biological processes such as the adjustment of digestive system functionality, female pregnancy, signal transduction, synaptic transmission, heart rate and blood pressure modulation, sleep, memory, and cognitive function. Even more, it possesses psychological effects by shaping emotion, social and romantic bonding, empathy, trust, and attachments.

Oxytocin also regulates reproductive functions, the birth process by stimulating the uterine muscles, lactation by inducing the contraction of myoepithelial cells in the milk ducts, and maternal and paternal behavior [132]. The oxytocin receptor is expressed in the hypothalamus and brainstem and in brain areas that process olfaction such as olfactory nuclei and piriform cortex, amygdala, nucleus accumbens, anterior pituitary, insula, and striatum [135].

On the other hand, cortisol represents a steroid hormone produced by the adrenal glands [136], thus being the final compound that results from the hypothalamic-pituitary-adrenal circuit. At the time stressor factors are taking over a person, his nervous system prepares an adequate activation response [137]. Concurrently, the HPA axis secretes high quantities of cortisol in order to adjust the body's functions. After the threat has passed, the axis proceeds by resetting its activity and reverting to its normal tone [138]. Nevertheless, if the stress exceeds and the body is stuck in a prolonged perceived threat situation, where large amounts of hormones are secreted, in the end, it will lead to a less reactive hypothalamic-pituitary-adrenal axis [139]. Furthermore, if the axis fails to revert to its standard function, unusual cortisol amounts can be found in individuals suffering from posttraumatic stress disorder [140]. As a consequence, we ask ourselves if it is possible to make a biological marker out of cortisol when it comes to people diagnosed with this disorder.

In general, soon afterwards a distressing experience (so-called “phasic” reactions), the production of cortisol is usually increased, a fact noticed in warriors who were attacked [141] and in women who suffered from rape [142]. On the contrary, baseline 24 h cortisol elimination was at a low point in individuals diagnosed with stress disorder in comparison with controls [143]. This can result from a higher epinephrine and cortical receptivity, acknowledging that in PTSD exists an enhanced dexamethasone suppressibility [144] or a lowered responsiveness of the adrenal cortex [145]. Another research study found a higher level of stress hormone in collected urine from a twenty-four-hour period in groups focusing on battle warriors suffering from PTSD [146], in females with stress disorder derivative from sexual assault during childhood [147], and in diagnosed minors subjected to physical abuse [148].

When it comes to oxytocin, the number of receptors and the blood concentration of this neurotransmitter may vary from one individual to another in response to an array of factors, as well as conditions of stress or distress. It was recently revealed in studies of psychopathology that trauma [149] and severe PTSD [150], as well as major depression disorder in women [151], are linked to extremely increased or fluctuating oxytocin levels, whereas insufficient oxytocin is associated with schizophrenia [152]. It is also known that the way oxytocin responds is partly modulated by the secretion of cortisol, which plays a fundamental role in the biomechanics of stress involving the hypothalamic-pituitary-adrenal axis [153]. However, in individuals diagnosed with PTSD, the system that involves the stress hormone is presumably impaired [154].

Oxytocin dysregulation that foregoes traumatic experiences could amplify the reaction of the central stress response system when dealing with maltreatment and the development of PTSD [155]. Both components mentioned above may work together and increase the cumulative burden of ongoing stress, ultimately triggering and setting the ground for stress-related illnesses.

Besides the aforementioned HPA axis, another stress system is represented by the locus coeruleus (LC) and norepinephrine pathways, which can globally modulate alert and arousal states, as the LC is engaged in times of acute or chronic distress. These two main circuits have been proven to link early-life trauma and PTSD [156].

Therefore, studies that examined the hypothalamic-pituitary-adrenal axis' standard activity in individuals diagnosed with posttraumatic stress disorder have not proven decisive, varying from a lower secretion of cortisol in some cases to a higher one in other cases.

Moreover, another study focused on neurobiological reactions when confronted with white noise and battle cries, where veterans diagnosed with posttraumatic stress disorder displayed increased cortisol amounts in comparison to the control group (of those who served in the military but had no prior diagnosis of PTSD) and controls from the general population [157]. However, there is one thing to ponder: the cortisol evaluation period (a single time prior the experiment and another time postexposure) has been improper to determine standard extent and cortisol response to distress. Therefore, it was deemed inconclusive if the high levels illustrated an increased arousal response or an anticipative reaction induced by the battle sound effects and white noise.

Furthermore, research on animal models reveals that elevated levels of glucocorticoid hormones can cast a shadow on memory functions, as prolonged distress impairs performance and cognitive capabilities by inducing a certain degree of vulnerability and susceptibility to the hippocampus (mediated by glucocorticoid receptors (GCR)) [158].

There are reasons to believe that individuals suffering from stress disorder report a heightened receptiveness of GCR in the hippocampal area [159]. If this is the case, exposure to stress might result in profound deterioration of explicit memory (episodic and sematic) in comparison to controls. That would eventually justify the fact that people diagnosed with stress disorder display impaired cognition and mind tasks, such as difficulties in acquiring fresh information, gaps in the autobiographical memory, and memory loss and dissociative amnesia [160].

In another study, women diagnosed with PTSD displayed remarkably high glucocorticoid amounts in comparison to molested females without the stress disorder when confronted with individualized stories of childhood mistreatment, with the highest peaks occurring in the process and a short time later after being exposed to narratives of traumatic experience. While recovering, glucocorticoid levels in the posttraumatic stress disorder category became notably low, comparable to those who did not suffer from this disorder [161]. In tune with previous research displaying lowered or adequate basal cortisol amounts, the discoveries can imply there are enhanced cortisol levels after being subjected to distress.

Other interesting findings were revealed in a study conducted by Dr. Young and Dr. Breslau, who focused on PTSD and its biological correlations [162]. It was shown that the category of people diagnosed with this disorder displayed remarkably elevated catecholamine amounts in comparison to the category of individuals subjected to distressing events and the category without any exposure. On the contrary, for mean cortisol levels, there were no significant differences across groups. Women who suffered from major depressive disorder in addition to PTSD displayed remarkably elevated glucocorticoid amounts in comparison to females diagnosed with one or the other psychiatric problem [162].

## 8. Conclusions

Posttraumatic stress disorder (PTSD) represents a mental health issue that develops after experiencing or witnessing terrifying situations, becoming especially relevant as the ubiquity of this ailment rises with its multifaceted psychosomatic comorbidities. Severe stress, which overcomes adequate response from the patient's side and generates disturbances, perturbations, and pathogenic effects, together with genetics and epigenetics subsequently leads to traumatic stress or psychological trauma.

PTSD is regarded as one of the most complex disorders, influenced by a series of biological, social, and psychological factors. It is also strongly connected to other psychiatric pathologies, such as massive depression disorder, anxiety, impulse control condition, suicide, and drug addiction, while also linked to various physical health diseases and complaints, including neurological, cardiovascular, endocrine, and gastrointestinal ailments.

PTSD aftermath comes forth from a wider network represented by disorders such as the metabolic syndrome, irritable bowel syndrome, bacterial overgrowth syndrome, gut microbiome alterations, hematopoiesis imbalance, or mechanisms/molecules like inflammation, oxidative stress, oxytocin, and cortisol.

Childhood adversities pave the way for psychological problems and psychiatric illnesses later on. Molecules such as cortisol and oxytocin play a significant role in emotional adjustment. Events that profoundly impact one's life during childhood or adolescence can alter and modify oxytocin levels in regions such as the hypothalamus and the amygdala, which are specialized in the production of oxytocin and emotional regulation.

It is wise to have a holistic approach and to perceive this syndrome intertwined with pathologies that arise from the digestive tract, incriminating factors such as the human microbiome and its potential to act as a mastermind inside the body, both as a healer and a disruptor or a trigger to various distresses (such as IBS, obesity, diabetes, and enteral infections). It is noteworthy to observe how the bacterial population impacts general health when it comes to different systems or organs and also that an imbalance of the beneficial/harmful bacteria can tip the scale in favor of a positive or negative outcome.

Equally important is the interwoven nature among metabolic disorders, obesity, or even hematological alterations and PTSD, suggesting there are common underlying neuroendocrine alterations that can enhance the likelihood of a general disturbance of metabolism or just some metabolic changes due to the experienced traumatic event. Processes such as heightened autonomic activity, glucocorticoid activation (by setting a scene for dyslipidemia and weight gain, thus leaning towards a higher risk for metabolic syndrome), immunological dysregulation, and behavioral factors are just a few incriminated links between these disorders.

Furthermore, as stated before, the cerebrum, having an elevated energy demand and requiring 1/5 of total O_2_ supply, becomes extremely vulnerable to the oxidation process, especially when it is imbalanced. That essential factor contributes to cerebral biochemical impairment (unrestricted production of ROS, elevated levels of RNS, altered levels of antioxidant glutathione, and reduced production and performance of major antioxidant molecules, thus leading to cellular damage, early instated senescence, and also neurodegeneration). This phenomenon together with an increased level of inflammation (through its proinflammatory cytokines and T regulatory cells) is described in several neuropsychiatric illnesses, and PTSD makes no difference.

In light of current events, the number of individuals diagnosed with posttraumatic stress disorder is envisioned to increase dramatically in the near future, as PTSD represents a serious health matter, which drives the research forward by innovating new techniques, fulfilling the need to further understand this disorder and its underlying mechanisms. In this regard, new developments and improved treatment intervention techniques are expected to benefit the scientific society and, more so, the sufferers, their close ones, and the general population altogether.

## Figures and Tables

**Table 1:** Behavior and somatic adjustment in the stress process [8].

Adjustment of behavior	Adjustment of somatic processes
Modifying the behavior according to the situation at hand	Redirecting the energetic force of the systems involved according to their necessity
Altered sensory threshold	Directing the needed compounds (O_2_, carbohydrates, lipids, proteins, vitamins, water, and minerals) towards CNS and other involved areas
Sharpened memory and sensation	Modified cardiac activity; elevation of blood pressure, heart rate, and force of cardiac contraction
A high sense of arousal and awareness	Elevated respiratory rate
Increased cognitive functions, attentiveness, and concentration	Increased gluconeogenesis+lipolysis
Suppression of behaviors related to nutrition (obtaining/consuming food)	Removing toxic compounds
Inhibition of reproductive functions	Freezing reproductive and growth axes
Inhibition of gastric motility; greater movement in the colon	Limiting the reaction to stress
Limiting the reaction to stress	Containment of inflammatory/immune response
Stress-induced analgesia	Release of norepinephrine and epinephrine
